# The potential role of auditory prediction error in decompensated tinnitus: An auditory mismatch negativity study

**DOI:** 10.1002/brb3.1242

**Published:** 2019-03-20

**Authors:** Mehrnaz Mohebbi, Ahmad Daneshi, Abdoreza Asadpour, Samer Mohsen, Mohammad Farhadi, Saeid Mahmoudian

**Affiliations:** ^1^ ENT and Head & Neck Research Center and Department, The Five Senses Institute Hazrat Rasoul Akram Hospital, Iran University of Medical Sciences Tehran Iran; ^2^ Department of Electrical Engineering Sharif University of Technology Tehran Iran; ^3^ Department of Otolaryngology, Faculty of Medicine Damascus University Damascus Syria; ^4^ Department of Otorhinolaryngology Hannover Medical University (MHH) Hannover Germany

**Keywords:** change detection, habituation, mismatch negativity, prediction error, sensory memory, tinnitus

## Abstract

**Introduction:**

Some tinnitus subjects habituate to their tinnitus but some others do not and complain of its annoyance tremendously. Normal sensory memory and change detection processes are needed for detecting the tinnitus signal as a prediction error and habituation to tinnitus. The purpose of this study was to compare auditory mismatch negativity as the index of sensory memory and change detection among the studied groups to search for the factors involving in the perception of tinnitus and preventing habituation in decompensated tinnitus subjects.

**Methods:**

Electroencephalography was recorded from scalp electrodes in compensated tinnitus, decompensated tinnitus, and no tinnitus control subjects. Mismatch negativity was obtained using the oddball paradigm with frequency, duration, and silent gap deviants. Amplitude, latency, and area under the curve of mismatch negativities were compared among the three studied groups.

**Results:**

The results showed lower mismatch negativity amplitude and area under the curve for the higher frequency deviant and for the silent gap deviant in decompensated tinnitus group compared to normal control and compensated tinnitus group.

**Conclusions:**

This study revealed a deficit in sensory memory and change detection processing in decompensated tinnitus subjects. This causes persistent prediction errors; tinnitus signal is consistently detected as a new signal and activates the brain salience network and consequently prevents habituation to tinnitus. Mismatch negativity is proposed as an index for monitoring tinnitus rehabilitation.

## 1 INTRODUCTION

Tinnitus is the conscious perception of a sound in the head or ears without an external physical sound source (Møller, 2003). While tinnitus is not annoying for some subjects and they habituate to the tinnitus easily, it is very bothersome for some others and affects them emotionally and psychologically (Andersson, 2002; Dauman, Tyler, & Aran, 1992; Kaltenbach, 2006; Tyler, 2006). In the modern medical approach to tinnitus, two distinct types of tinnitus can be recognized. Decompensated tinnitus is defined as a complex psychosomatic process in which the person suffers considerably from tinnitus and does not habituate to it. In this context, mental and emotional factors affect the perception of tinnitus. Psychological symptoms such as difficulty in falling asleep or insomnia, aggression, concentration difficulties, anxiety, depression, and even suicidal thoughts are common in these subjects (Axelsson & Ringdahl, 1989; Lenarz, 1998; Malakouti, Mahmoudian, Alifattahi, & Salehi, 2011; Stobik, Weber, Münte, Walter, & Frommer, 2005; Tyler & Baker, 1983). Tinnitus is considered compensated when the person hears this phantom sound but habituates to it and does not essentially feel affected by it, or only complains in specific situations such as quiet environments, stressful situations, physical tension (Stobik et al., 2005).

Generally, tinnitus can be a result of deafferentation in auditory input but the exact pathophysiology of tinnitus has remained unclear (De Ridder, Vanneste, Weisz et al., 2014). Different mechanisms have been suggested for tinnitus pathophysiology (Preece, Tyler, & Noble, 2003), such as the increased spontaneous activity in the auditory cortex (Roberts, 2018), tonotopic map reorganization (Eggermont, 2006), enhanced neural synchrony (Shore, Roberts, & Langguth, 2016), noise cancelling (Rauschecker, Leaver, & Mühlau, 2010), and the involvement of the efferent auditory system (Geven, Köppl, de Kleine, & van Dijk, 2014). The recent models have introduced the activation of overlapped cooperate networks between auditory and nonauditory areas of the brain (Farhadi et al., 2010; Vanneste & De Ridder, 2012). Tinnitus‐related networks have been introduced as the interactions among perception, salience, and distress networks with auditory and somatosensory cortex as well as memory areas (Noreña & Farley, 2013). Regardless of tinnitus generation source, all recent models agree that brain central processing contributes to the perception of tinnitus (Eggermont, 2003; Lockwood, Salvi, & Burkard, 2002). Perception is an active process which requires both bottom‐up, that is, sensory and top‐down, that is, prediction processing. The perception of tinnitus has been always a matter in question.

De Ridder, Vanneste, Weisz et al., 2014 proposed that the Bayesian brain model of perception applies to the concept of tinnitus. The Bayesian model of perception suggests that the brain actively searches for sensory information in the environment to reduce sensory uncertainty by filling in the missing information (Friston, 2010). The brain keeps a prior template of what it is going to encounter in memory. It compares the new sensory input to the prior template and if it is different from what is expected, a prediction error occurs and the error is processed. This prediction error signal becomes the input for the subsequent processing level (Ramnani, 2006). This template is updated by continuous sampling from the environment. In this model, perception is the consequence of top‐down information processing, depending on what is expected in the sensory input (bottom‐up processing) and relying on what is stored in memory (De Ridder, Vanneste, & Freeman, 2014).

It has been suggested that the auditory mismatch negativity (MMN) is the neurophysiologic index of change detection process and identifies the prediction error caused by comparison to prior expectations (Näätänen, Kujala, & Winkler, 2011). MMN is a change specific component considered as an objective indicator of the brain's automatic change detection and auditory sensory memory (Ulanovsky, Las, & Nelken, 2003). It compares the neural reaction caused by the deviant stimulus with the sensory memory trace of the preceding standard stimuli, even in impaired consciousness and the absence of attention. MMN is evoked by any discriminable change in auditory stimulation. It reveals the process of automatic change detection when an internal representation of the environment conflicts with the incoming sensory stimulus (Escera, Alho, Winkler, & Näätänen, 1998). It helps to reveal the perceptual aspects of auditory processing.

According to Hallam's theory, tinnitus is a habituation deficit which is a central top‐down mechanism (Hallam, Jakes, & Hinchcliffe, 1988). Normal sensory memory and change detection processes are needed for detecting the tinnitus signal as a prediction error and providing habituation to tinnitus (Kisley, Noecker, & Guinther, 2004; Lijffijt et al., 2009). Normal sensory memory is needed for normal function of sensory gating and habituation mechanisms (Kisley et al., 2004; Lijffijt et al., 2009; Mohebbi, Farhadi, Daneshi & Mahmoudian, in press). It has been proposed that constant updating of the tinnitus percept from memory may prevent habituation to tinnitus (De Ridder et al., 2006). Sensory memory deficit was reported in tinnitus subjects comparing MMN in tinnitus subjects to normal controls (Mahmoudian et al., 2013; Weisz, Voss, Berg, & Elbert, 2004). It has been reported that N1 response as an index of late sensory gating was less decreased in amplitude when repetitive auditory stimuli were presented in decompensated tinnitus subjects (Walpurger, Hebing‐Lennartz, Denecke, & Pietrowsky, 2003). It means that there is a less habituation to irrelevant stimuli. What is still largely unknown is the perception of tinnitus and factors that involve in preventing habituation in decompensated tinnitus subjects. MMN may reveal the mechanisms underlying tinnitus perception and habituating to it.

A few studies have investigated MMN in tinnitus; among them some have looked at MMN using auditory stimuli with different deviants (Mahmoudian et al., 2013; Weisz et al., 2004). To our knowledge, no studies have used frequency of MMN stimuli in the range of tinnitus pitch.

This study aimed to search for the possible causes of habituation deficit in decompensated tinnitus subjects in the context of Bayesian perception model. The main purpose of this study was to investigate the differences in sensory memory and change detection processes among the studied groups. We hypothesized that sensory memory as indexed by MMN deficits in decompensated tinnitus subjects compared to compensated tinnitus subjects and normal controls; this deficit prevents normal prediction error occurring in the perception of tinnitus and thus prevents habituating to tinnitus.

## 2 METHODS AND MATERIALS

### 2.1 Subjects

The study subjects who voluntarily participated in the study consisted of three groups: 20 compensated tinnitus, 20 decompensated tinnitus, and 20 normal hearing subjects without tinnitus as the control group. They were all native Persian speaking and right‐handed as assessed by the Edinburgh Handedness Inventory (Oldfield, 1971). They were between 18 and 59 years old and had no current or past substance abuse or dependency, no neurological illness, no brain injury. A steady‐state tinnitus was present for more than 6 months in tinnitus subjects with the pitch range of 6–9 KHz. They were referred by the tinnitus clinic of Rasool‐e‐Akram general hospital. Otoscopy revealed healthy ear canals and tympanic membranes, and tympanometry showed bilateral normal external and middle‐ear functions in all subjects. Pure tone audiometry was performed for frequencies of 125 Hz to 12 KHz. The behavioral pure tone thresholds were ≤20 dB HL in octave frequencies of 250–2,000 Hz, and less than 40 dB HL in frequencies of 4,000 and 8,000 Hz in both ears. Inclusion criteria for compensated tinnitus subjects were: no complaint of tinnitus annoyance, visual analog scale (VAS) of less than 3 (out of 10) for tinnitus loudness, annoyance, and awareness, a score of less than 16 in tinnitus handicapped inventory (THI) (Mahmoudian et al., 2011) and less than 30 in tinnitus questionnaire (TQ) (Hallam et al., 1988). Inclusion criteria for decompensated tinnitus subjects were as follows: sever complaint of tinnitus annoyance, VAS of more than 7 for tinnitus loudness, annoyance, and awareness, a score of more than 58 on THI and more than 60 on TQ. Space‐occupying lesions such as acoustic neuroma were ruled out by magnetic resonance imaging (MRI). None of the tinnitus subjects received any treatments on the brain or ears before or after the onset of tinnitus and had no alcohol/drug abuse during the 3 months before the experiment day. The human subjects’ permission and a written informed consent were received from all subjects according to the Declaration of Helsinki principles. The ethical review board of Iran University of Medical Sciences (IUMS) approved the study procedure with the code of IR.IUMS.REC 1396.29494.

### 2.2 Tinnitus assessment

To determine the characteristics of subjective tinnitus before including subjects in the study, psychoacoustic tinnitus assessment including pitch matching of tinnitus (PMT), loudness matching of tinnitus (LMT), minimal masking level (MML), and residual inhibition (RI) were performed in a sound‐treated room using an audiometer calibrated according to ANSI 2006 (Madsen Astera^2^).

In pitch‐ and loudness‐match tests, sounds were presented to contralateral ear to the tinnitus ear in unilateral subjects or contralateral to the most bothersome tinnitus ear in bilateral subjects. LMT was obtained in all frequencies of test tones (from 125 Hz to 12 kHz) in order to provide equal sensation level for all frequencies in performing PMT. Pitch matching will be more accurate by having loudness matching of all tones because loudness perception changes by changing frequency. Tone levels were increased in 1‐dB step levels starting just below the hearing threshold level until the subjects stated that the sound level was equal to tinnitus loudness. LMT was reported in dB sensation level (dB SL). We used a two‐alternative forced‐choice method to obtain PMT (Vernon & Fenwick, 1984). Different pairs of tones typically in multiples of 1 KHz were presented at LMT of each tone. Subjects were instructed to identify which tone best matched the pitch of their tinnitus. Octave confusion test was performed at PMT. The LMT obtained at tinnitus pitch was considered as LMT. MML and RI (using acoustical stimuli) were measured in the ipsilateral ear. Narrow band noise (NBN) of the center frequency of PMT was used in measuring MML and RI (Feldmann, 1971). NBN hearing threshold was obtained at all frequencies used for measuring PMT and increased in 1‐dB increments in each frequency. Subjects were asked to report the moment that they did not perceive their tinnitus. Acoustical RI was measured using NBN at the corresponding frequency of PMT presented at an intensity of 15 dB above MML (15 dB SL) for 1 min. NBN was delivered binaurally for bilateral tinnitus and monaurally for unilateral tinnitus.

### 2.3 Procedure

Tinnitus subjects filled VAS, Persian TQ (Daneshi, Mahmoudian, Farhadi, Hasanzadeh, & Ghalebaghi, 2005), and Persian THI (Mahmoudian et al., 2011). To record electroencephalography (EEG), subjects were seated on a comfortable chair in an electromagnetic proof and sound‐treated room. Their head, neck, and back were supported with pillows to reduce muscle contractions. We instructed them to be relaxed, not to pay attention to the auditory stimuli, stay awake, and avoid blinks and any eye and body movements during recording but not to try too hard to suppress eye blinks. Once the EEG cap fixed on the scalp, hairs under each electrode site were wiped using a blunt needle to expose the skin under the hair and a conducting gel was injected to each electrode site so that it contacted with the skin under it. Subjects watched a subtitled silent movie (Planet Earth, BBC Documentary production, 2008) played on the front screen to keep them alert and help them ignore the stimuli during the experiment. EEG recording session including preparation and recording lasted for about 35 min.

### 2.4 Stimuli

**Figure 1:**
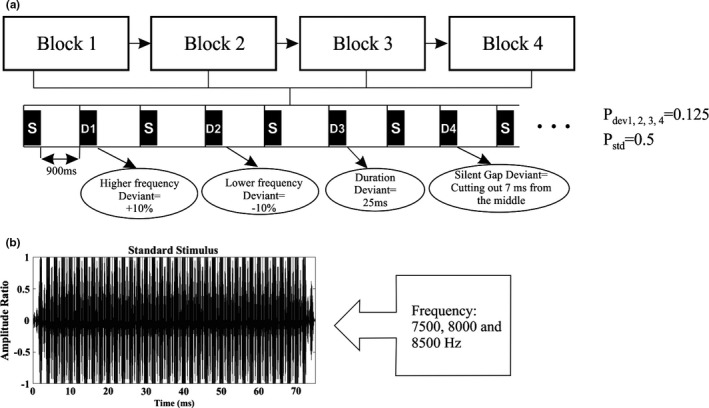
Diagram illustrating multifeature mismatch negativity (MMN) paradigm and stimuli features: (a) The sequence of standard and deviant stimuli in each block. (b) The waveform of the standard stimulus composed of three sinusoidal tones

The proposed paradigm by Näätänen, Pakarinen, Rinne, & Takegata, 2004 was used to obtain MMN. This paradigm considerably shortens the recording time while enables to record multiple types of MMN. In this paradigm, each deviant is presented after a standard stimulus, meaning that the deviants occur with the probability of 50% relative to standards. Each stimulus was 75 ms in duration with 5 ms rise and fall time. Standard stimuli consisted of three sinusoidal tones with frequencies of 7,500, 8,000, and 8,500 Hz. According to the study of Mahmoudian et al., 2013, three types of deviates which showed significant differences between tinnitus and normal subjects were used: frequency, duration, and silent gap. Frequency deviants composed of three tones which half of them were 10% higher than the standards (8,250, 8,800 and 9,350 Hz) and the other half were 10% lower than the standards (6,750, 7,200 and 7,650 Hz). In duration deviants, the duration of stimuli was 50 ms lower than the standards (15 ms plateau and 5 ms rise and fall time). Silent gap deviants consisted of 7 ms silent (with 1 ms rise and fall time) in the middle of the standard stimulus. The stimuli paradigm is shown in Figure 1. The stimuli were constructed digitally using MATLAB platform. The oddball paradigm was designed in four blocks. In each block, there were 91 stimuli with 50% standards and 50% deviants. Also, the first 10 stimuli in each block were only standards and deviants were presented pseudo‐randomized within a block so that there was always a deviant between two standards and two standards never followed each other. Interstimulus interval was 900 ms. Stimuli were presented at an intensity of 85 dB SPL through two speakers with the 45° angle and 1.5 meters distance in front of the subjects. Stimuli presentation lasted for 6 min.

### 2.5 EEG recording

A 64‐channel BRAIN QUICK LTM (Micromed, Italy) was used to record electrical brain activities referenced to the tip of the nose. Twenty nine scalp sites (FP1, FPz, FP2, F7, F3, Fz, F4, F8, FT7, FC3, FCz, FC4, FT8, T7, C3, Cz, C4, T8, TP7, CP3, CPz, CP4, TP8, P3, Pz, P4, POz, M1, and M2) were selected according to the International 10‐10 system to place Ag/AgCl electrodes. The ground electrode was on Fz (Oostenveld & Praamstra, 2001). An electrode below and the other at the outer canthus of the left eye recorded electrooculogram (EOG) activity. Electrodes impedance was under 10 KΩ during recording. The sampling rate for digitalization of EEG signals was 1,024 Hz. EEG was filtered by an online 0.4–200 Hz band‐pass filter and 50 Hz notch filter for removing power line interference. Moreover, digital events from presentation software were received by a custom‐designed microcontroller device. It transformed them to be compatible with the trigger signal and marked the events on the computerized EEG recordings.

### 2.6 EEG data preprocessing

We used the EEGLAB 11.02 toolbox and MATLAB software for analyzing EEG data offline (Delorme & Makeig, 2004). An offline 1–20 Hz band‐pass filter was used to filter EEG data. A microcontroller device generated trigger signals on continued EEG. Epochs were extracted from EEG data according to the trigger signals. All data were baseline corrected for amplitude measurement using the nearest extremum voltage level to the MMN peak. EEG data were decomposed into independent components using Independent Component Analysis (ICA). Then it was checked for eye blinks, electrocardiographic (ECGs), and other muscular components by visual inspection. If Epochs amplitudes exceeded 50 μV, they were rejected from subsequent processing. Epochs were averaged in 50 ms prestimulus to 900 ms poststimulus for standard and each type of deviant stimuli separately. The first 10 standards of each block were rejected from the averaging.

### 2.7 EEG data analysis

Responses to the standard stimuli were subtracted from each type of deviant to calculate MMN waveforms. Because the largest negative MMN peak is typically found in F3, F4, Fz, FC3, FC4, FCz, and Cz electrodes, they were selected as region of interest (ROI) and amplitude, latency, and area under the curve were calculated over these sites. The most negativity of averaged MMN over ROI in a time window of 100–250 ms poststimulus was used to label and calculate amplitudes and latencies. The area under the curve feature was calculated from that part of the curve between the nearest extremum to the peak of MMN and the baseline which considers the baseline. Polarity reversals at channels M1 and M2 occurring at the 100–250 ms poststimulus period confirmed MMN waveforms validity. The grand average waveforms and isopotential topographic maps were obtained.

### 2.8 Statistical analysis

The Statistical Package for Social Science (SPSS V.16; Chicago, United States) was used to perform all statistical analyses. A *t *test or one‐way ANOVA was used to compare demographic characteristics. To compare MMN features among the studied groups, one‐way ANOVA was calculated for each feature. A repeated measures ANOVA was computed to evaluate the effects of deviant types on MMN features within each group. Additionally, to find significant differences between the factor deviant, the Bonferroni adjustment was utilized to perform multiple comparisons of MMN features for all deviants within each group.

## 3 RESULTS

### 3.1 Demographics

A total of 60 subjects participated in this study. They were 30 males and 30 females (10 males and 10 females in each group). The mean age had no significant differences among the three studied groups as shown in one‐way ANOVA test. Also, one‐way ANOVA test for hearing thresholds in frequencies of 250, 500, 1,000, 2,000, 4,000, 6,000, and 8,000 Hz did not show significant differences among the three groups. Tinnitus laterality in compensated tinnitus group consisted of six subjects in the right ear, seven subjects in the left ear, and seven subjects with bilateral tinnitus. Tinnitus laterality in decompensated tinnitus group included five subjects in the right ear, eight subjects in the left ear, and seven subjects with bilateral tinnitus. Tinnitus duration, PMT, and LMT were not significantly different between compensated and decompensated tinnitus group as indicated by *t *test. A Mann–Whitney test was performed to compare scores for THI, TQ, and VAS for loudness, VAS for annoyance, and VAS for awareness between compensated and decompensated tinnitus groups. It showed that THI, TQ, and VAS scores were significantly higher in decompensated tinnitus group compared to compensated tinnitus group. Demographic characteristics are indicated in Table 1.

**Table 1:** Demographic characteristics of the studied groups

	Normal control		Compensated tinnitus		Decompensated tinnitus		*F*_(2,57)_	*t*	*p*
	Mean	±*SD*	Mean	±*SD*	Mean	±*SD*			
Age	40.05	11.55	44.35	11.49	42.35	11.31	0.70	—	0.49
Pure Tone Thresholds									
250	10.25	8.02	11.50	6.30	12.75	7.51	0.58	—	0.56
500	13.25	9.63	14.75	8.34	12.00	6.15	0.56		0.57
1,000	17.25	11.17	15.75	8.47	14.50	6.04	0.48		0.61
2,000	19.00	12.73	18.25	11.61	17.00	7.32	0.17		0.84
4,000	14.75	8.95	18.00	12.60	21.00	10.07	1.72		0.18
6,000	18.50	11.48	20.00	9.59	22.00	8.33	0.63		0.53
8,000	25.00	10.76	21.25	9.30	24.00	11.98	0.65		0.52
Tinnitus duration	—		61.45	45.73	70.05	82.12	—	−0.40	0.68
PMT LMT	— —		7.50 6.75	1.60 2.73	7.60 7.10	1.42 2.78	—	−0.20 −0.40	0.83 0.69
VAS for loudness	—		2.45	0.82	8.20	1.23	—	−17.26	0.00**
VAS for annoyance	—		2.10	0.96	8.60	1.18	—	−18.97	0.00**
VAS for awareness	—		1.95	0.68	8.60	1.14	—	−22.31	0.00**
TQ	—		26.70	5.79	66.85	10.84	—	−14.59	0.00**
THI	—		19.60	5.93	74.90	11.81	—	−18.70	0.00**

Note

The statistical significance is marked with asterisks.

PMT: pitch matching of tinnitus; LMT: loudness matching of tinnitus; VAS: visual analog scale; TQ: tinnitus questionnaire; THI: tinnitus handicapped inventory.

**p < 0.01.

### 3.2 MMN differences among groups

**Figure 2:**
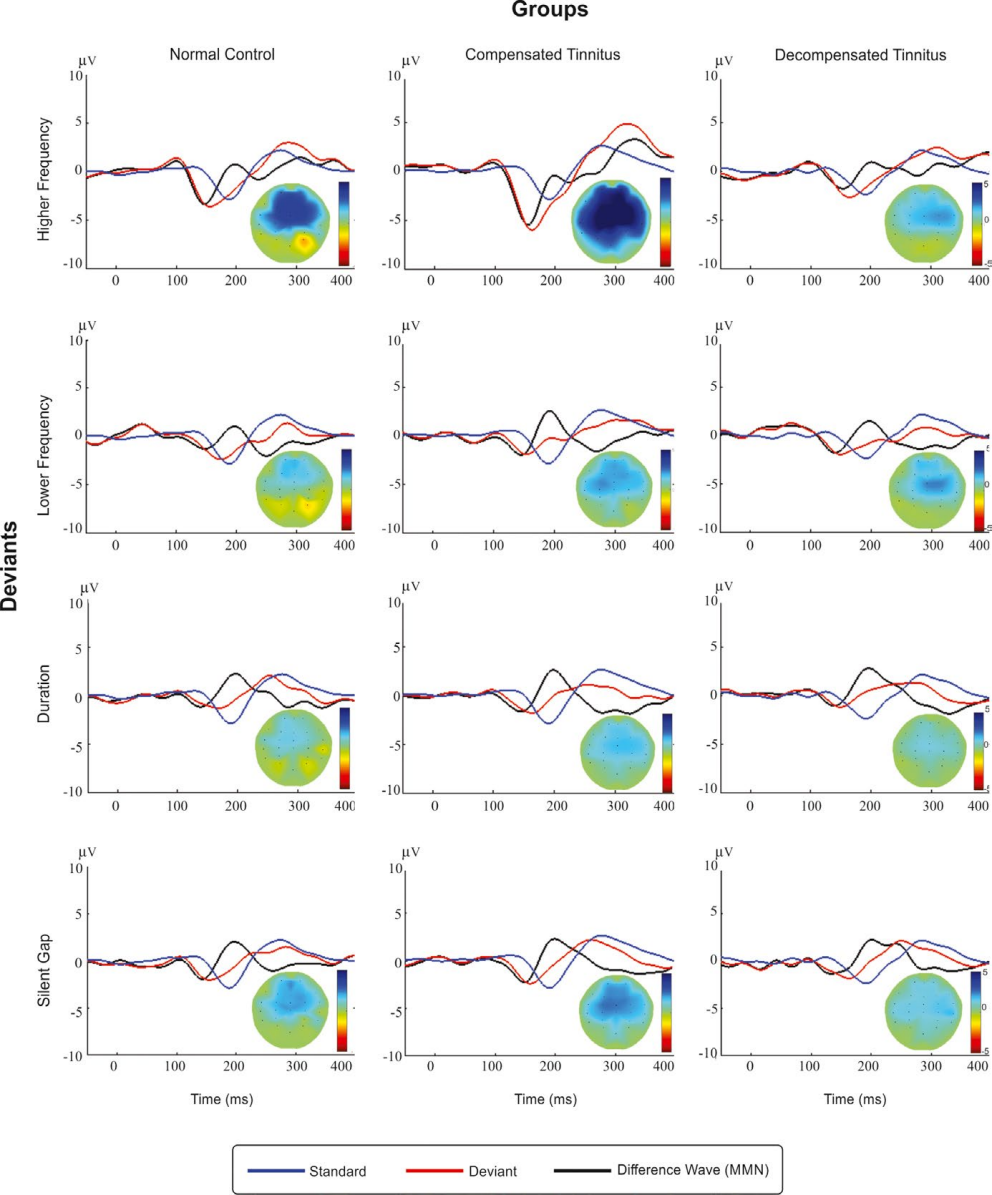
The grand mean average of auditory mismatch negativities (MMNs) recorded in the studied groups. Event‐related potentials to standards (blue line) and deviants (red line) are demonstrated for a frontocentral region of interest (ROI: F3, F4, Fz, FC3, FC4, FCz, and Cz) for each type of deviant, averaged across all subjects. In addition, difference waves are given for a frontocentral ROI (black line). Topographies at MMN peak maximum at ROI are illustrated for each group and for each type of deviant

The grand average of event‐related potentials (ERPs) for the standards and deviants was extracted. The grand average of MMN waveforms was calculated by subtracting ERPs of deviants from standards. ERPs elicited by standards and deviants along with their associated MMN waveforms are shown in Figure 2. They were obtained for higher frequency, lower frequency, duration, and silent gap deviants.

**Figure 3:**
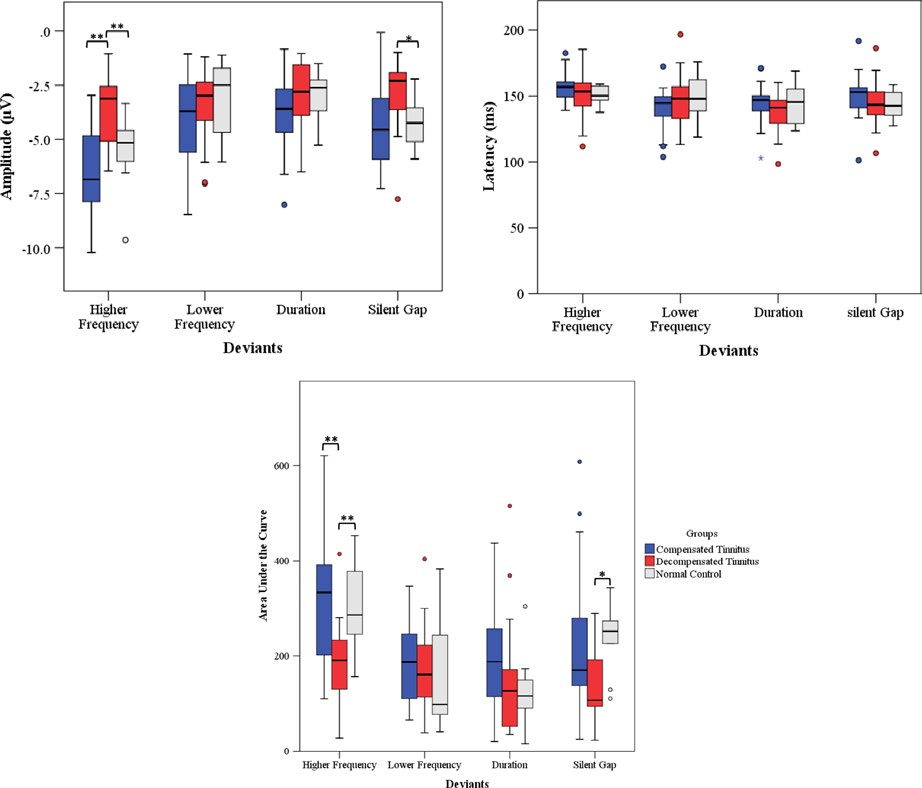
The boxplot showing pairwise comparisons of amplitude, latency, and area under the curve of mismatch negativity (MMN) for each type of deviant among the three studied groups. The statistical significance is marked with asterisks: *p < 0.05; **p < 0.01

A one‐way ANOVA was performed to compare the means of MMN amplitude, latency, and area under the curve among the three studied groups (Table 2). The results showed that MMN amplitude and area under the curve for higher frequency and silent gap deviants significantly differed among the three studied groups. No differences were seen for latencies. Tukey posthoc test revealed that MMN amplitude and area under the curve for higher frequency deviant and for silent gap deviant were significantly larger in decompensated tinnitus group compared to normal control and compensated tinnitus group (Figure 3).

**Table 2:** Statistical results for one‐way ANOVA comparing means of latency, amplitude, and area under the curve for MMNs of different deviants in the three studied groups

Deviants	Mismatch negativity (MMN) features	Normal controls		Compensated tinnitus group		Decompensated tinnitus group		*F*_(2,57)_	*p*
		Mean	±*SD*	Mean	±*SD*	Mean	±*SD*		
Higher Frequency	Amplitude	−5.49	1.68	−6.64	2.08	−3.78	1.55	13.41	0.00**
	Latency	150.27	7.75	158.14	10.67	152.35	18.03	1.97	0.14
	Area Under the Curve	310.32	85.04	323.49	147.46	185.96	84.18	9.96	0.00**
Lower Frequency	Amplitude	−3.04	1.65	−4.02	1.95	−3.39	1.66	1.60	0.21
	Latency	149.64	16.45	140.45	17.43	146.74	19.74	1.36	0.26
	Area Under the Curve	159.51	114.38	182.30	84.90	167.60	88.43	0.28	0.75
Duration	Amplitude	−3.00	1.14	−3.70	1.66	−2.90	1.47	1.85	0.16
	Latency	143.60	15.22	145.83	15.37	137.44	15.63	1.63	0.20
	Area Under the Curve	121.39	78.99	184.50	95.77	146.13	119.99	2.02	0.14
Silent Gap	Amplitude	−4.20	1.16	−4.03	2.13	−2.85	1.53	4.07	0.02*
	Latency	143.98	10.99	151.44	16.68	144.92	17.74	1.38	0.25
	Area Under the Curve	242.57	62.74	210.25	154.88	137.13	71.94	5.49	0.007**

Note

The statistical significance is marked with asterisks.

*p < 0.05.

**p < 0.01.

### 3.3 MMN differences within groups

A one‐way repeated measures ANOVA was performed in each group to compare MMN features for all types of deviants. The results in the normal control group showed that there was a significant main effect of amplitude (*F*(3, 57) = 24.49, *p* = 0.00), latency (*F*(3, 57) = 3.03,*p* = 0.03), and area under the curve (*F* (3, 57) = 23.97, *p* = 0.00) on types of deviants. In compensated tinnitus group, there was a significant main effect of amplitude (*F*(3, 57) = 24.75,*p* = 0.00), latency (*F*(3, 57) = 6.59, *p* = 0.01) and area under the curve (*F*(3, 57) = 10.57, *p* = 0.00) on types of deviants. In decompensated tinnitus group, there was a significant main effect only of latency (*F*(3, 57) = 3.28, *p* = 0.02) on types of deviants.

**Figure 4:**
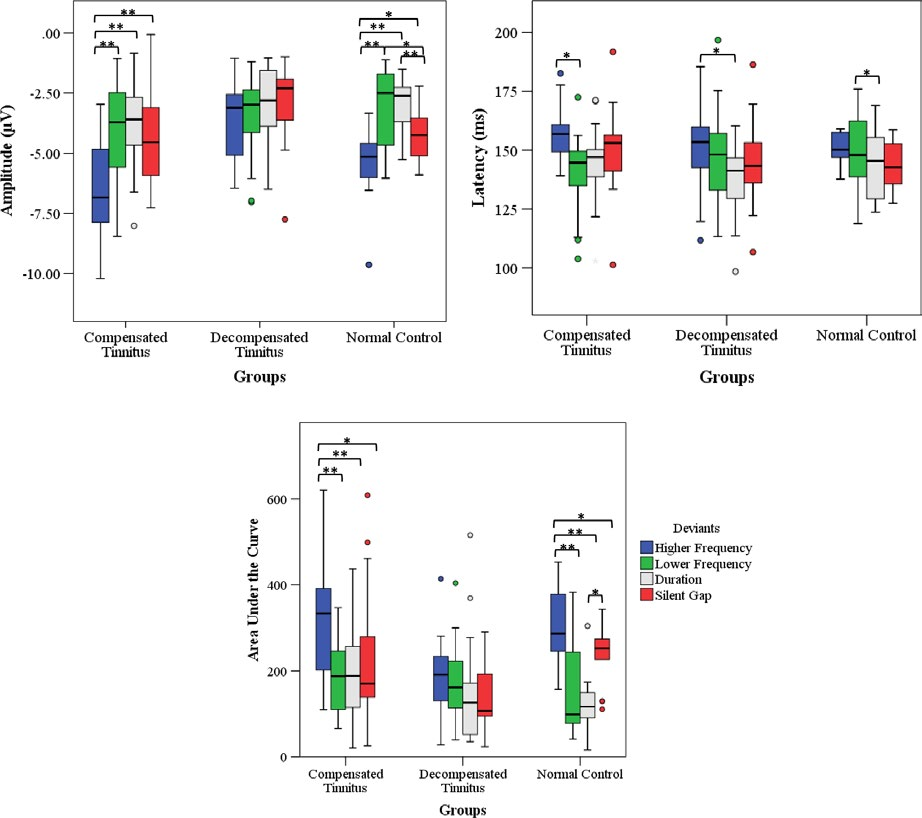
The boxplot showing comparisons between the mismatch negativity (MMN) for different types of deviants in each studied group. It is clear that comparisons in the decompensated tinnitus group did not indicate the significant differences in amplitude and area under the curve. The statistical significance is marked with asterisks: *p < 0.05; **p < 0.01

Bonferroni posthoc tests revealed significant differences in pairwise comparisons for MMN amplitude and MMN area under the curve (higher frequency–lower frequency, higher frequency–duration, higher frequency–silent gap, silent gap–lower frequency, and silent gap–duration) in the normal control group. Posthoc tests in compensated tinnitus group showed significant differences in pairwise comparisons for MMN amplitude and area under the curve (higher frequency–lower frequency, higher frequency–duration, and higher frequency–silent gap) and for MMN latency (higher frequency–lower frequency). While in decompensated tinnitus group, Bonferroni posthoc test indicated significant differences only in MMN latency (high frequency–duration). The comparisons are indicated in Figure 4.

## 4 DISCUSSION

This study compared MMN features among compensated tinnitus subjects, decompensated tinnitus subjects, and normal controls. We found that MMN amplitude and area under the curve for higher frequency and silent gap deviants were significantly lower in decompensated tinnitus subjects compared to the other studied groups.

### 4.1 MMN differences among groups

A few studies have investigated MMN in tinnitus, among them a few looked at MMN using auditory stimuli with different deviants and frequencies. To our knowledge, this is the first study comparing MMN among compensated, decompensated tinnitus subjects, and normal controls and also using auditory stimuli in the frequency range of tinnitus pitch. MMN reveals the process of automatic change detection in sensory stimulus (Escera et al., 1998; Escera, Yago, Corral, Corbera, & Nuñez, 2003) by comparing the deviant stimulus with the sensory memory trace of the preceding standard stimuli. To date, the mechanisms underlying tinnitus and its persistence remain unclear but it is accepted that the perception of tinnitus is a result of aversive brain central processing (Eggermont, 2003; Lockwood et al., 2002).

MMN deficit in tinnitus subjects have been reported in previous studies and our results are consistent with them. Weisz et al., 2004 reported that the tinnitus subjects demonstrated significant abnormalities in MMNs specific to frequencies located at the audiometrically normal lesion‐edge as compared to healthy controls. Mahmoudian et al., 2013 indicated lower MMN amplitude and area under the curve for frequency, duration, and silent gap deviants in tinnitus subjects compared to normal controls. They concluded a possible deficit in auditory preattentive change detection processing. Our results agree with them in significant amplitude and area under the curve differences for high frequency and silent gap deviants. The main difference between our study and Mahmoudian et al., 2013 was that we divided tinnitus subjects into two groups of compensated and decompensated, so to be able to investigate tinnitus mechanism more precisely. Moreover, we used auditory stimuli with frequency adjusted to the subjects’ tinnitus pitch range. It is expected that auditory event‐related potentials characteristics differ when auditory stimuli correspond with tinnitus pitch or edge frequency of hearing loss (Sereda, Adjamian, Edmondson‐Jones, Palmer, & Hall, 2013). Limited studies in tinnitus subjects are designed in this way. However, pitch matching data suggest that most patients do not have a pitch‐match frequency just below the maximum hearing threshold loss frequency. This argues against the brain reorganization model in most subgroups of tinnitus (Pan et al., 2009).

In the current study, MMN amplitudes and area under the curves in all studied groups seem to be lower than the previous studies which used low‐frequency stimuli (Mahmoudian et al., 2013; Weisz et al., 2004). Wunderlich & Cone‐Wesson, 2001 reported that the amplitude of MMN decreased as the frequency of auditory stimuli increased in normal healthy subjects. This might be due to the facts that low‐frequency sounds activate a larger portion of the basilar membrane and that neural generators for lower frequencies are positioned at the higher level on the surface of the cortex compared to high‐frequency generators in the cortex; so larger amplitudes of MMN are recorded (May et al., 1999). Consistent with previous studies, our results confirm that the detection of differences in high‐frequency stimuli is more difficult than the low‐frequency stimuli. It may be related to the decrease in sensation level at high frequencies (Wier, Jesteadt, & Green, 1977). So we suggest that using auditory stimuli matching the tinnitus pitch may better reveal the processing deficit in MMN.

Using high‐frequency stimuli, we found that MMN amplitude and area under the curve for higher frequency and silent gap deviants were significantly lower in decompensated tinnitus subjects compared to the other studied groups. The lower amplitude and area under the curve of MMN for silent gap deviant in decompensated tinnitus group may be due to gap detection deficit in tinnitus. Studies on gap detection indicated a consistent deficit in gap processing in tinnitus subjects (Fournier‐Viger, Faghihi, Nkambou, & Nguifo, 2012). Our result on MMN supports this hypothesis that tinnitus may fill in the silent gaps and makes it difficult for the auditory cortex to detect the silent gap (Mahmoudian et al., 2013). Another recent study reported that deficit in gap processing in tinnitus subjects is linked to deficient timing cues and deficient temporal discrimination caused by processing alterations in tinnitus (Ku et al., 2017). Whereas we tried to minimize the effect of hearing loss in the studied subjects, but we cannot ignore the presence of up to 40 dB HL hearing loss in frequencies of 4,000 and 12,000 Hz in some subjects. Therefore, the deficit in gap processing in tinnitus subjects may relate to the probable hearing loss, as gap detection problems are evident in many with hearing loss (Tyler, Summerfield, Wood, & Fernandes, 1982).

However, what can be concluded is that the presence of a decompensated tinnitus may cause a deficit in the perception of the silent gap. The gap detection deficit might be an index of abnormal cortical auditory processing in tinnitus.

The lower amplitude and area under the curve of MMN for higher frequency deviant in decompensated tinnitus group may be due to enhanced frequency resolution and frequency discrimination in tinnitus subjects. Thai‐Van, Micheyl, Moore, and Collet (2003) suggested that persistent auditory stimulation results in enhancing frequency discrimination and resolution. It has been shown that frequency discrimination training results in cortical reorganization and increases the numbers of neurons respond to the trained frequency (Recanzone, Schreiner, & Merzenich, 1993). The significant result in higher frequency deviant may be due to this fact that frequency discrimination training resulted from persisting tinnitus sound had modulated the brain synchronous activities. It has been reported that tinnitus subjects with a mild hearing loss at tinnitus pitch have a more amplitude‐dependent N1–P2 response in the tinnitus frequency relative to controls (Kadner et al., 2002). The enhancement of frequency discrimination and resolution due to tinnitus results in facilitated comparisons between the deviant and standard stimuli and the decrease of MMN amplitudes. Our results for duration deviant were near to be significant. They might statistically be significant if the number of subjects was increased.

According to the Bayesian brain model, the brain relies on internal probabilities to adjust function in situations of uncertainty (Friston, 2010; Ostwald et al., 2012). In this model, the incoming sensory input is compared with the existing prior knowledge in memory to predict. Unitary sensory memory representations are then created and used to form predictions and create auditory objects (Winkler & Czigler, 2012; Winkler & Schröger, 2015). The incoming sensory input is compared with the existing internal memory representation and if they were different, a prediction error occurs. The brain only allows the prediction errors to pass onto the next level of processing. This Bayesian prediction has been verified by electrophysiology. MMN (Näätänen et al., 2011) and P300 (Polich, 2007) are known as neurobiological indicators associated with Bayesian brain hypothesis (Baldi & Itti, 2010; Itti & Baldi, 2009). The N100 is an index of sound detection and sensation (Parasuraman & Beatty, 1980; Winkler, Tervaniemi, & Näätänen, 1997). MMN reflects the automated change detection based on prediction error processing; however, the P300 might involve attention orientation toward deviant stimuli and context perception (King, Gramfort, Schurger, Naccache, & Dehaene, 2014; Polich, 2007; Schwartze, Tavano, Schröger, & Kotz, 2012). MMN detects the difference between the repetitive standard stored in the sensory memory and the oddball deviant. It works based on detecting the changes between the incoming stimuli and the existing memory representations. So, it can show the processing according to the Bayesian model.

Consistent with Durai, O'Keeffe, and Searchfield (2018), our results provide evidence that sensory memory is occupied by the intrinsic tinnitus signal, so the change detection mechanism is not able to retain the incoming signal to use it for comparison and detect the changes. Constant updating of tinnitus percept from memory as a result of deficient sensory memory prevents habituation (De Ridder et al., 2006). Normal sensory memory and change detection processes are needed for detecting the tinnitus signal as a prediction error and habituating to tinnitus. Using the Bayesian model, we propose that abnormal sensory memory function prevents prediction error caused by the tinnitus signal. The tinnitus signal cannot be maintained to the existing prior template in memory, so it is persistently detected as a prediction error and passes the tinnitus signal onto the next level of processing. This is why tinnitus signal is consistently detected as a new signal and activates the brain salience network and prevents habituating to tinnitus. This indicates that the dishabituation to tinnitus in decompensated tinnitus subjects is not only related to characteristics of the sensory input, that is, the bottom‐up processing, but rather a top‐down processing associated with detection of a mismatch between the internal expectations and the incoming information. It can be hypothesized that sensory memory and prediction error mechanisms in compensated tinnitus subjects are similar to normal subjects but deficits of sensory memory and prediction error prevent habituating to tinnitus in decompensated tinnitus subjects.

De Ridder, Vanneste, Weisz et al. (2014)) applying the Bayesian model to tinnitus proposed that tinnitus perception results from the underlying neural reorganization of the tonotopic areas caused by auditory deafferentation. They believe that auditory deafferentation leads to topographically restricted prediction errors, although the absence of an expected stimulus induces a cortical prediction error signal. If uncertainty cannot be reduced by getting information from the adjacent cortical regions, the missing information can be recalled from the memory stored in the parahippocampal region (De Ridder, Vanneste, & Freeman, 2014). The involvement of the parahippocampus in tinnitus might be related to the constant updating of the tinnitus percept from memory, thereby preventing habituation (De Ridder et al., 2006).

Although the hypothesis proposed by De Ridder, Vanneste, Weisz et al. (2014) can apply to tinnitus perception, but considering that not all the tinnitus subjects have necessarily hearing loss and consequently, auditory deafferentations; we suggest that prediction errors caused by the tinnitus signal is related to the deficient brain comparator in top‐down processing, so it cannot identify tinnitus as a repetitive signal in the memory. As a result, tinnitus is continuously updated from memory regardless of its origin (being due to peripheral deafferentation or cortical regions). It is detected as a prediction error and is sent to higher order processing of the brain and finally prevents habituation. This is why decompensated tinnitus subjects have the best MMNs in the frequency range of tinnitus pitch confirmed by higher frequency deviant MMN, but it has significantly lower amplitudes in other deviants, meaning that the process of comparing deviants to standards is not working properly as shown in Figure 3 waveforms. From the figure, it is clear that although MMN for higher frequency deviants showed desirable amplitudes and waveforms in all groups the response to standard and the deviant significantly decreased in decompensated tinnitus groups. It reveals that although the comparisons of standard stimuli to deviants are happening but possibly due to difficulty of access to memory during the comparisons, the response to this deviant decreased compared to the other groups.

Neuroimaging studies supported this hypothesis in tinnitus showing that dorsal anterior cingulate cortex and insula that are brain regions related to MMN and P300 are activated during filling in mechanisms to restore missing information in tinnitus subjects (Shahin, Bishop, & Miller, 2009). The neural networks, activated when a stimulus is predicted closely, are similar to memory retrieval processes (Albright, 2012). Persisting tinnitus causes involvement of memory networks (De Ridder et al., 2006; Mahmoudian et al., 2013). The presence of tinnitus led to the involvement of working memory which can hypothetically affect the accuracy of predictive processing (Durai et al., 2018). Previous studies have shown that MMN is elicited by auditory cortex, frontoparietal brain areas, the dorsal anterior cingulate cortex, insula and dorsolateral prefrontal cortex, (i.e., salience network) (Molholm, Martinez, Ritter, Javitt, & Foxe, 2005; Takahashi et al., 2013). Interestingly, many of the brain areas involved in tinnitus overlay the MMN‐related regions. It can be concluded that lower MMN amplitudes and area under the curve in decompensated tinnitus subjects may be due to abnormal activity of salience network. The activity of other annoyance and awareness neural networks that emphasize on thoughts and emotions are also emphasized in the Tinnitus Primary Functions Questionnaire (Tyler et al., 2014). Also, it can be concluded that the presence of tinnitus cannot be a problem by itself but the involvement of other nonauditory neural networks like salient network causes tinnitus annoyance and awareness.

### 4.2 MMN differences within groups

The results of pairwise comparisons among MMN for different deviants showed that the number of significantly different MMNs pairwise comparisons in decompensated tinnitus group was less than the other groups. This supports the observed decrement of MMN amplitude and area under the curve in decompensated tinnitus group seen in between‐group comparisons. This finding emphasizes that decompensated tinnitus subjects may have deficient auditory discrimination and sensory memory in the central auditory processing, regardless of deviant type. The pairwise comparisons among MMN for different deviants in each feature revealed that the best types of deviants for eliciting MMN with strong amplitude and area under the curve are associated with higher frequency and silent gap deviants. Mahmoudian et al. (2013) also reported the silent gap deviant as one the best deviants for evoking MMN. The longer latency of MMN for higher frequency deviant in both tinnitus groups may be related to this fact that the frequency of this deviant was matched with tinnitus pitch. This might cause the auditory processing time to become longer for that deviant.

Within‐group differences in MMNs for various deviants may also suggest that different neural populations establish the generation of MMNs as suggested by Giard, Perrin, Pernier, and Bouchet (1990). They reported that frequency, intensity, and duration of auditory stimuli have separate neural representations in sensory memory. Though, further studies are needed using procedures with high‐spatial resolution in order to determinate the localization of the MMN generators and their characteristics in different types of deviants in normal and tinnitus subjects. However, because the difficulty of deviant detection was not perceptually the same among all the deviant types, they cannot be directly compared. This does not entirely prevent comparisons, but we need to be careful when making interpretations.

### 4.3 Topographic maps

Visual inspection of topographic maps also showed that MMN amplitudes in decompensated tinnitus group are smaller in each deviant compared to the other groups. They confirm the statistical comparisons among the groups. It should be noticed that although the maps are visually informative and are congruent to the statistical results, we must be careful in interpreting them because they show the distribution of MMNs only on ROI electrodes.

## 5 LIMITATIONS

There were some limitations in the current study which cannot be neglected. It was very difficult to find tinnitus subjects with entirely intact hearing thresholds and tinnitus subjects with moderate high‐frequency hearing loss were enrolled in the study. Although there was no significant difference in hearing thresholds in the tinnitus groups, it should be reported that some MMN changes could have been as a result of hearing loss. Homogeneous subjects are needed in a group to obtain much‐validated results. In this study, wide range of subjects’ age and laterality of tinnitus might have been limiting factors that could affect the interpretation of results regarding the central auditory processing. Also, since the sensitivity of tinnitus assessment tools such as THI has been challenged for categorizing compensated versus decompensated tinnitus (Tyler, Noble, & Coelho, 2006), it is suggested that the findings of this study be confirmed by future studies. We recommend further studies to consider these factors in designing their studies. However, the current findings suggest a significant auditory processing deficit in decompensated tinnitus subjects and the results can provide a reference point for future studies.

## 6 CONCLUSION

This study proposed a deficit in sensory memory processing and prediction error in decompensated tinnitus subjects as revealed by MMNs for high frequency and silent gap deviants. Abnormal sensory memory function caused by the tinnitus induces persistent prediction errors. The tinnitus signal cannot be maintained as an existing prior template in memory, so it is persistently detected as a prediction error and the tinnitus signal is passed into the higher brain regions for next levels of processing. This is why tinnitus signal is consistently detected as a new signal and activates the brain salience network and prevents habituating to tinnitus. The enhancement of frequency discrimination and resolution due to tinnitus results in facilitated comparisons between the deviant and standard stimuli and the decrease of MMN amplitudes. MMN can be an index for monitoring tinnitus rehabilitation.

## CONFLICT OF INTEREST

Authors disclose no conflict of interest in financial or personal relationships.

## AUTHOR CONTRIBUTIONS

It is confirmed that the manuscript has been drafted and read critically and final approved by all abovementioned authors. All the authors certify that they contributed in the study procedure and manuscript preparation as follows:

- Mehrnaz Mohebbi contributed in idea and designing the study, acquisition, analysis, or interpretation of data and drafting the primary article. This study was a part of her Ph.D. project.
- Professor Ahmad Daneshi and Professor Mohammad Farhadi contributed in conception and design of the study from the aspects of clinical representations and interpretation of results.
- Abdoreza Asadpour as the signal processing expert engineer contributed in technical designing and production of stimuli, EEG and ERP analysis, and technical interpretation of ERPs.
- Dr. Samer Mohsen contributed in acquisition, analysis, or interpretation of data.
- Professor Saeid Mahmoudian contributed in the idea, conception, designing the study from the aspect of neuroscience and interpretation and justification of final results.

Also, all the authors agree to be accountable for all aspects of the study in ensuring that questions related to the accuracy or integrity of any part of the study are appropriately investigated and resolved.
